# BACE2 variant identified from HSCR patient causes AD-like phenotypes in hPSC-derived brain organoids

**DOI:** 10.1038/s41420-022-00845-5

**Published:** 2022-02-02

**Authors:** Juan Luo, Hailin Zou, Yibo Guo, Ke Huang, Elly Sau-Wai Ngan, Peng Li

**Affiliations:** 1 Scientific Research Center, The Seventh Affiliated Hospital of Sun Yat-sen University, Shenzhen, 518107 Guangdong People’s Republic of China; 2 Department of Surgery, Li Ka Shing Faculty of Medicine, University of Hong Kong, Pokfulam, Hong Kong; 3 Guangdong Provincial Key Laboratory of Digestive Cancer Research, The Seventh Affiliated Hospital of Sun Yat-sen University, No. 628 Zhenyuan Road, Shenzhen, 518107 Guangdong People’s Republic of China

**Keywords:** Induced pluripotent stem cells, Neurodegeneration

## Abstract

β-site APP-cleaving enzyme 2 (BACE2) is a homolog of BACE1, which is considered as the most promising therapeutic target for Alzheimer’s disease (AD). However, the expression and functional role of BACE2 in central nervous system (CNS) remain obscured. Previously, we identified several BACE2 rare variants in Hirschsprung disease (HSCR) patients and proved that BACE2-mediated APP cleavage might represent a novel HSCR pathogenesis mechanism in enteric nervous system. Here, we validated that these HSCR-associated BACE2 variants were loss-of-function mutations. Using the human pluripotent stem cell (hPSC)-derived brain organoids (BOs), we further demonstrated that BACE2 was mainly expressed in the ventricular zone and cortical plate of BOs, and its expression level was gradually increased along with the BO maturation. Functionally, we found that the BOs carrying the BACE2 loss-of-function mutation (*BACE2*^*G446R*^) showed greater apoptosis and increased levels of Aβ oligomers compared to the control BOs, resembling with the AD-associated phenotypes. All these phenotypes could be rescued via the removal of APP protein in *BACE2*^*G446R*^ BOs. Furthermore, rather than *BACE2*^*G446R*^, *BACE2*^*WT*^ overexpression in BOs carrying the APP Swedish/Indiana mutations attenuated the AD-associated phenotypes, including Aβ accumulation and neuronal cell death. Taken together, our results unravel that BACE2 can protect the neuronal cell from apoptosis caused by Aβ accumulation, and the deficiency of BACE2-mediated APP cleavage may represent a common pathological mechanism for both HSCR and AD.

## Introduction

AD is the most common neurodegenerative disease leading to dementia, and it is clinically manifested with progressive memory loss and cognitive dysfunction. Amyloid-β (Aβ) accumulation-induced neuritic plaques in the brain is the characteristic AD neuropathology, which eventually causes neuronal cell death [1]. Aβ peptide is generated from the sequential cleavage of amyloid precursor protein (APP) by β- and γ-secretases. The β-site cleavage of APP within the luminal domain releases a C-terminal fragment (C99), which can be further cleaved by γ-secretase to produce the N-terminal fragment Aβ [2, 3]. Mutations happened in APP protein, which facilitates the β- or γ-site cleavage to produce Aβ, will result in an early-onset AD [4–6].

BACE1 was initially identified as a β-site APP-cleaving enzyme in vivo to generate Aβ, owing that genetic deletion of BACE1 in an AD mouse model has abolished the Aβ production [7, 8]. Therefore, inhibition of BACE1 activity has been considered as a promising strategy for AD drug development. Indeed, BACE1 inhibitor Verubecestat (MK-8931) has previously been shown to efficiently reduce the Aβ levels in AD animal models and patients [9]. BACE2 is a homology of BACE1 and initially considered to be another β-secretase [2, 10, 11]. However, unlike BACE1, which is highly expressed in the CNS, there is a moderate amount of BACE2 expression in the human brain. Conversely, BACE2 is prominently found in peripheral tissues to execute other functions, including the colon, kidney, and pancreas [12]. For example, BACE2 could cleave the pro-proliferative plasma membrane protein TMEM27 in pancreas, and thereby impacting the β cell mass and functions [13, 14]. In addition, BACE2-mediated PMEL protein cleavage in pigment cells was essential for melanosome formation, and genetic deletion of BACE2 in mouse caused coat color defects [15]. In CNS, emerging evidences revealed that BACE2 functioned like an α-secretase, rather than a β-secretase in APP processing [16, 17], thereby competing with BACE1 to cleave APP and inhibiting Aβ generation. However, its impact on AD pathogenesis or therapy is still obscured.

Many HSCR-associated mutations were found to affect the CNS and some HSCR patients also have CNS disorders [18]. We previously have performed the whole-genome sequencing analyses of patients with HSCR and identified the dysregulation of BACE2-mediated APP cleavage as novel pathogenesis in enteric nervous system [19]. In this study, we further confirmed that the BACE2 variants identified from HSCR patients were loss-of-function mutations, and the hPSC-derived BOs carrying these variants exhibited AD-like phenotypes, including Aβ accumulation and neuronal cell death. Furthermore, BACE2 overexpression in BOs with APP Swedish/Indiana mutations attenuated the AD-associated phenotypes. Our findings thus unraveled that BACE2 played a protective role in neuronal cell death through inhibition of Aβ production and accumulation.

## Materials and methods

### Human PSC maintenance

Human ES cells (H1) or Control iPSCs (UE02302) used in this study were gifts from Dr Ke Huang (Guangzhou Institutes of Biomedicine and Health, China). The hPSCs were cultured on Matrigel (BD Biosciences, 354234)-coated plates with the defined medium mTeSR1 (Stemcell Technologies, 05850), and the culture medium was changed daily.

### Brain organoid induction

hPSC-based BOs were generated according to manufacturer’s instructions of the commercial STEMdiff™ Cerebral Organoid Kit (Stemcell Technologies). Briefly, on day 0, human PSC colonies were dissociated into single-cell suspension with Accutase and 1 × 10^4^ cells were then seeded into one well of a U-bottom ultra-low-attachment 96-well plate in EB formation medium supplemented with 10 μM Y27632. The EB formation medium was refreshed every other day. On day 5, the EBs were transferred to a 24-well low attachment plate in neural induction medium for another 3–5 days. On day 9, the EBs were embedded into 15 μl Matrigel and cultured in neural expansion medium for 3 days in a six-well low attachment plate. On day 12, the organoids were moved to maturation medium on an orbital shaker for 40–60 days. Subsequently, the organoids were directly collected for our experiments.

### Genome editing

The CRISPR-Cas9^D10A^ nickase-based genome editing system was used to generate the *APP* knockout iPS cell line as previously described [20]. Briefly, two single guide RNAs targeting the exons 3 of *APP* gene locus were created according to the gRNA cloning protocol. 4 million of iPS cells were transfected with gRNA constructs prepared and a GFP-fused Cas9^D10A^ nickase expression plasmid by electroporation using Nuclofector transfection kit (Lonza, VPH-5022). After transfection, the cells were seeded on Matrigel-coated plate with mTeSR1 medium for 48 h, and then the GFP-expressing cells were sorted into Matrigel-coated 96-well plate by fluorescence-activated cell sorter to get single cell. Single colony was formed ~7–14 days and then the single colony was passaged twice using ReLeSR^TM^ (StemCell Technologies, 05872) according to the manufacturer’s protocol. Subsequently, the genomic DNA was isolated and the targeted region of *APP* gene was PCR amplified and directly sequenced. The single colony with bi-allelic nonsense mutations was expanded and used for the follow-up assays.

### DNA constructs and lentivirus production and infection

The lentiviral expression constructs pEF1-MCS-3xFlag, subcloned with human *APP* (*APP*^*WT*^ or *APP*^*Swe/Idl*^) or *BACE2 (BACE2*^*WT*^ or *BACE2*^*G446R*^*)* were purchased from TranSheepBio company http://www.transheep.com/. For lentiviral production and infection, lentiviral plasmid (1.2 μg), together with 0.8 μg of packaging plasmid pSPAX2 (Addgene #12260) and 0.5 μg of envelope expressing plasmid (Addgene #12259) were transiently co-transfected into the 293T cells using the Lipofectamine 2000 reagent according to the manufacturer’s instructions. 48 h after transfection, the lentivirus supernatant was collected and filtered with 0.45 μm membrane filters (Millipore). Human PSCs were infected in the presence of 5 μg/mL polybrene and selected with 1 μg/mL puromycin for 72 h. The empty vector was used as a control in above-mentioned lentiviral transfection experiment.

### Reverse transcription and quantitative real-time PCR

Reverse transcription and qRT-PCR assays were conducted following previously described protocols [21, 22]. Real-time PCR analysis was performed using the BioRad machine with the SYBR Premix Ex Taq (TaKaRa). The oligo sequences of RT primers used in this study were listed in supplementary table 1.

### Western blotting

Human iPSCs/ESCs or BOs were lysed firstly using the RIPA protein lysis buffer (50 mM Tris-HCl, pH 7.5, 100 mM NaCl, 1% Tritone X-100, 0.1 mM EDTA, 0.5 mM MgCl_2_, Inhibitors of proteases, and phosphatases), and then we followed the methods previously described to do the following steps [21, 22]. The primary and secondary antibodies and corresponding dilution ratios used in this study were listed in supplementary table 2.

### Immunostaining

hPSC-based BOs were fixed firstly in 4% paraformaldehyde for 30 min then dehydrated with 30% sucrose in PBS at 4 °C. Subsequently, the organoids were embedded with optical cutting temperature (OCT) compound and frozen on dry ice. Frozen tissue was sectioned at 20 μm using a cryostat and collected on ultra-frosted glass microscope slides. For immunostaining, the sections were permeabilized in 0.25% Triton X-100 and blocked with 1% BSA in PBS for 1 h, followed by incubation with primary antibody overnight at 4 °C. The primary antibodies and dilutions used in this study were listed in Supplementary table 2. After washing with PBS three times on the second day, the samples were incubated with the appropriate secondary antibodies, conjugated with Alexa Fluor 594 (Molecular Probes) in PBS for 1 h at room temperature. Sections were then counterstained with 4, 6-diamidino-2-phenylindole dilactate (DAKO) for 15 min at RT following the method previously described. Images were captured using a Carl Zeiss confocal microscope (LSM 800).

### Data analysis

Statistical significance was determined by the unpaired Student’s *t* test. The *P*-value is indicated by asterisks in the Figures (*P < 0.05 and **P < 0.01). Differences of *P* < 0.05 and lower were considered statistically significant.

## Results

### HSCR-identified BACE2 variants are loss-of-function mutations

**Fig. 1:**
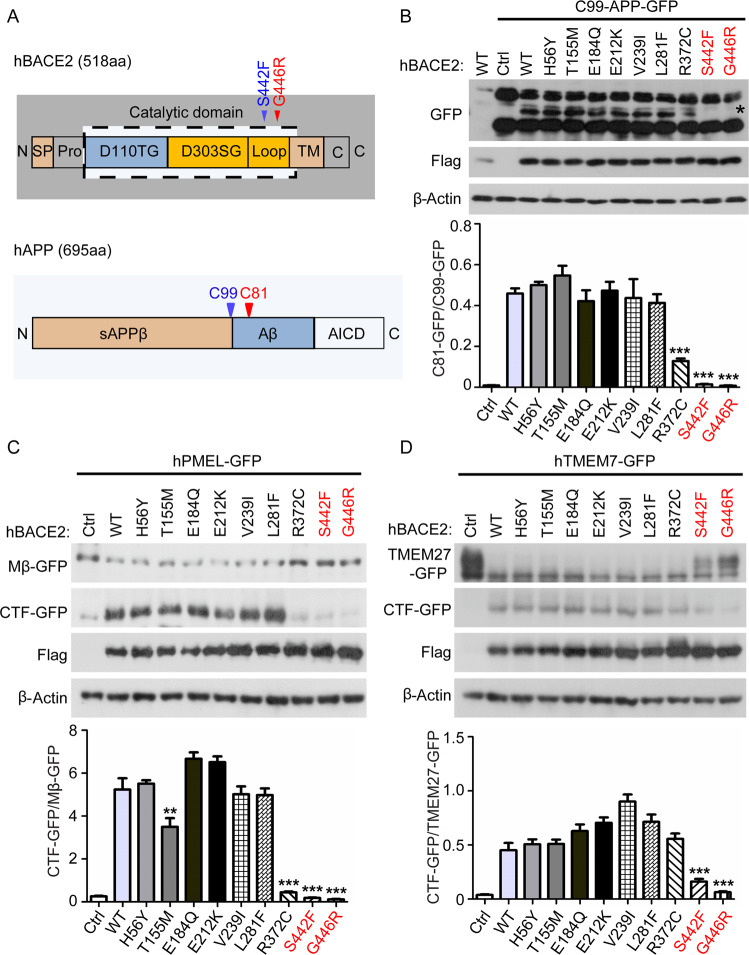
HSCR-identified BACE2 variants are loss-of-function mutations. **A** hBACE2 protein domain map with S442F and G446R mutations indicated, and hAPP protein domain map with C99 and C81 sites indicated. **B**–**D** Western blot analysis of the BACE2 enzymatic activity carrying the HSCR-identified variants according to the substrate-based cleavage efficiency, including C99-APP, hPMEL, and hTMEM7. The quantification data are shown as the mean ± S.D (*n* = 3). Statistically significant differences are indicated.

Previously, our whole-genome sequencing analyses of patients with HSCR-identified BACE2 as a genetic factor, which contributed to the disease development through mediating the APP cleavage in enteric nervous system [19]. Since *APP* is a well-studied AD-related gene, we aim to examine the effects of BACE2-mediated APP cleavage in CNS. Firstly, we tested the cleavage activities of all BACE2 variants identified in HSCR patients using the known substrates, including C99-APP, PMEL, and TMEM7. We overexpressed wild-type (WT) or Flag-tagged BACE2 variants together with GFP-tagged substrates at the C-terminal in 293T cells, and the protease activity of BACE2 was then monitored based on the production of C-terminal fragment using western blot analysis. As shown in Fig. 1, the BACE2 variants encoding R372C, S442F, and G446R dramatically inhibited their cleavage activity. In particular, the variants residing adjacent to the transmembrane domain of BACE2 (S442F and G446R), which may interfere with the membrane docking of BACE2, completely abolished the protease activity of BACE2 (Fig. 1A–D and S1A–C). These results are consistent with our conclusions previously described [19], and also indicated that the HSCR-identified BACE2 variants were loss-of-function mutations.

### Establishment of mature BOs with human PSCs

**Fig. 2:**
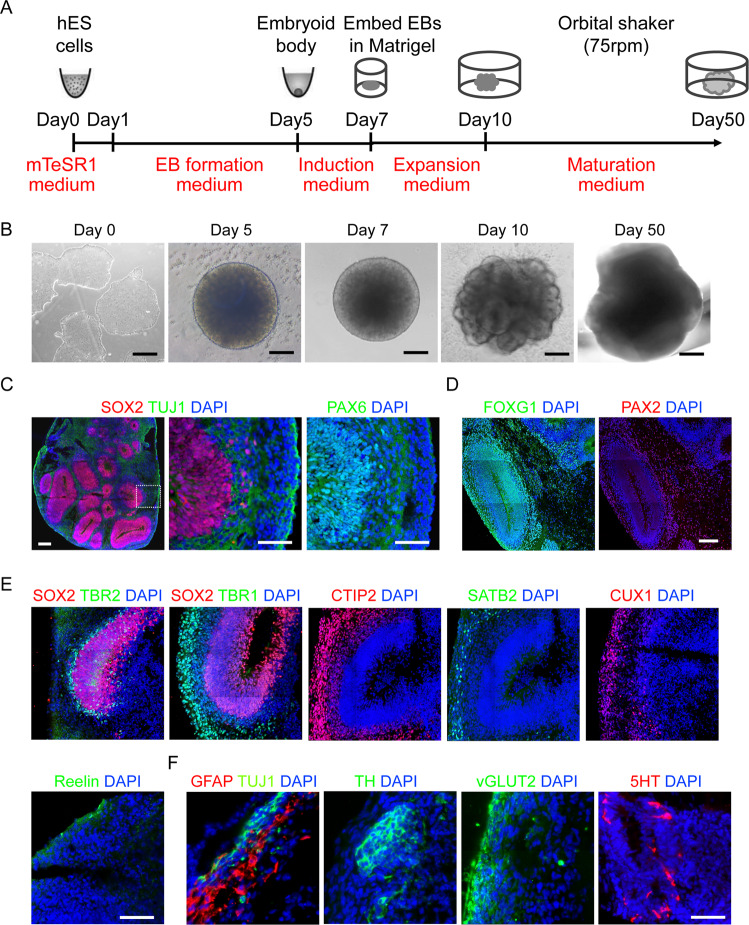
Establishment of mature BOs with hPSC. **A** A schematic overview of the procedures for generating BOs from hPSCs using the STEMdiff™ Cerebral Organoid Kit. **B** Representative images of hPSC-derived cells at the corresponding stages. **C** Representative images of the ventricular zone-like structure formed by new-born neurons (Tuj1^+^) and neural progenitor cells (SOX2 and PAX6^+^) in BOs at day 40. Scale bar: 100μm. **D** Representative images of the positive staining regions for forebrain (FOXG1) and hindbrain (PAX2) markers respectively. Scale bar: 100 μm. **E** Representative images showed the immunostaining of the six layers of cortical neurons at day 60. Scale bar: 100 μm. **F** Confocal images showed the positive staining regions for the astrocyte markers GFAP, and the expression of different subtypes of functional neuron markers, such as TH, 5-HT and vGLUT2. Scale bar: 100 μm.

To examine the impact of BACE2-mediated APP cleavage in the brain, we first developed a 3D BO model using hPSCs following an optimized protocol developed by Stemcell Technologies. In all, 1 × 10^4^ hPSCs were initially used to grow a cellular aggregate and form the embryoid body (EB) with 5 days, and then the EBs were transferred into the neural induction medium for 2 days, followed by expansion of neuroepithelia in expansion medium with a Matrigel scaffold for 3 days. Subsequently, the hPSC-derived BOs were transferred to an orbital shaker in maturation medium and maintained under rotary conditions for 40 days (Fig. 2A, B). On day 40, the organoids exhibited a typical dorsal forebrain region specification with a fluid-filled ventricle-like structure aligned with SOX2 and PAX6-positive neural progenitors in a ventricular/subventricular-like zone and TUJ1-positive neuroblasts in a cortical plate (Fig. 2C). Furthermore, staining for markers of the forebrain (FOXG1) and hindbrain (PAX2) revealed the specification of these regions, although they did not organize to form the overall structure seen in vivo (Fig. 2D). Subsequently, we also performed detailed expression analyses of markers for different neuronal subtypes and observed that the organoids expressed all the neuronal markers of six cortical layers, including deep layer cortical neuron markers TBR1 and CTIP2, upper layer cortical neuron marker SATB2, layer II/III markers CUX1, and Cajal-Retzius cell marker REELIN (Fig. 2E), which closely resembled with the developing human cortex. At day 60, we could also detect the marker expressions of different functional neurons and glia, including glutamatergic (vGLUT2), dopaminergic (TH), and serotonergic (5-HT) neurons, and GFAP + astroglia (Fig. 2F). All these results demonstrated the BOs could be developed successfully with cortical like organization composed of abundant mature neurons and astrocytes.

### BOs with BACE2 loss-of-function mutation exhibit AD-like phenotypes

**Fig. 3:**
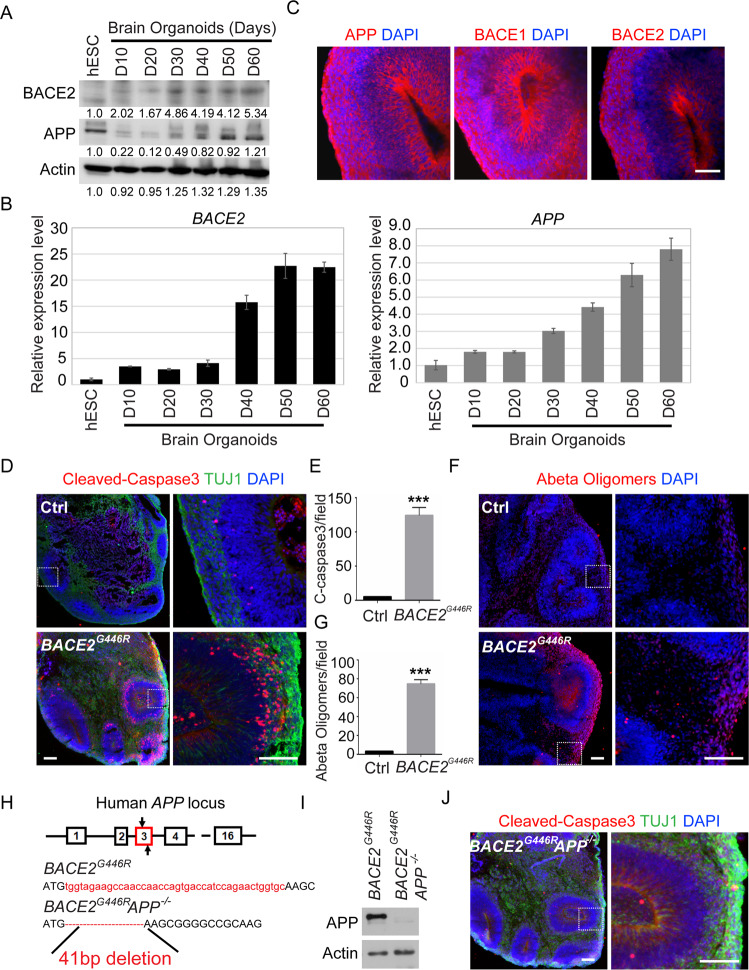
BOs with BACE2 loss-of-function mutation exhibit AD-like phenotypes. **A** Western blot analyses of total proteins from hPSC-derived BOs from day 0-60 using the indicated antibodies. **B** qRT-PCR analysis of the mRNA levels of *APP* and *BACE2* in hPSC-derived BOs from day 10 to 60. The quantification data are shown as the mean ± S.D (*n* = 3). **C** Confocal images showed the expression and distribution of BACE1, BACE2 and APP in hPSC-derived BOs on day 40. Scale bar: 100 μm. **D**, **E** Representative images and quantitation of cellular apoptosis evaluated by immunostaining of Cleaved-caspase3, the quantitation data were from 3 BOs per cell line. Scale bar: 100 μm. **F**, **G** Representative images and quantitation of Aβ accumulation evaluated by immunostaining of Aβ oligomers, the quantitation data were from 3 BOs per line. Scale bar: 100 μm. **H**–**I**. Schematic of CRIPSR targeting exon 3 of *APP* gene to generate *BACE2*^*G446R*^/*APP*^*-/-*^ iPSC line from *BACE2*^*G446R*^ parental line. A pair of gRNAs targeting and putative cut sites of Cas9D10A are indicated. *BACE2*^*G446R*^/*APP*^*−/−*^ carries a 41 bp deletions in both alleles are confirmed by sanger sequencing and western blotting analysis. **J** Representative images showed the cellular apoptosis by immunostaining of Cleaved-caspase3 in *BACE2*^*G446R*^ and *BACE2*^*G446R*^/*APP*^*-/-*^ hPSC-derived BOs. Scale bar: 100 μm.

Then to determine the functional role of BACE2-mediated APP cleavage in the brain, we firstly analyzed the expression patterns of BACE2 and APP in different development stages of BOs from day 0 to day 60 through western blot and qRT-PCR assays. We observed that BACE2 was rarely detected, while APP was abundantly expressed in hPSCs; however, both of their expressions were gradually increased from day 10 to day 60 (Figs. 3A, B and S2A), indicating the potential roles of BACE2 and APP in BO derivation. Besides, immunostaining with APP, BACE1, and BACE2 antibodies revealed that BACE1, BACE2, and APP were mainly expressed in the ventricular zone and cortical plate, despite the BACE2 expression level was obviously lower than BACE1 and APP in these regions (Fig. 3C). Subsequently, we directly differentiated the control and *BACE2*^*G446R*^ hPSCs to grow BOs using the established organoid culture system previously described. The size and morphology of BOs were monitored from day 1 to 60, and there were no evident differences observed among them. Then, to evaluate the effect of BACE2-mediated APP cleavage on neuronal apoptosis in organoids, cleaved-caspase3 was analyzed by immunostaining at day 60 and we found the cleaved-caspase3 immunoreactivity was increased in the organoids with BACE2 loss-of-function mutation compared to the control group (Fig. 3D, E). Meanwhile, to investigate whether the neuronal apoptosis in *BACE2*^*G446R*^ organoids was caused by the loss of BACE2-mediated APP cleavage activity, immunostaining assay was performed using Aβ oligomer antibody and the results revealed that lots of Aβ oligomers were detected in *BACE2*^*G446R*^ organoids compared to control group (Fig. 3F, G), suggesting the alteration of BACE2-mediated APP cleavage in HSCR-associated BOs resulted in enhanced Aβ oligomer accumulation and induced the neuronal cell apoptosis, resembling with the AD-associated phenotypes. To further validate this conclusion, *BACE2*^*G446R*^ hPSCs were used to generate a double-mutant line (*BACE2*^*G446R*^*APP*^*-/-*^) using CRISPR/Cas9 technology (Fig. 3H, I). As anticipated, deletion of APP significantly improved the survival of neuronal cells in *BACE2*^*G446R*^ BOs, as evidenced by reduced cleaved caspase3 staining (Fig. 3J). Taken together, our data demonstrated that BACE2 could protect the neuronal cells in brain from undergoing apoptosis by properly processing APP and preventing the Aβ accumulation.

### BACE2 overexpression in BOs with family APP mutations attenuates AD-associated phenotypes

**Fig. 4:**
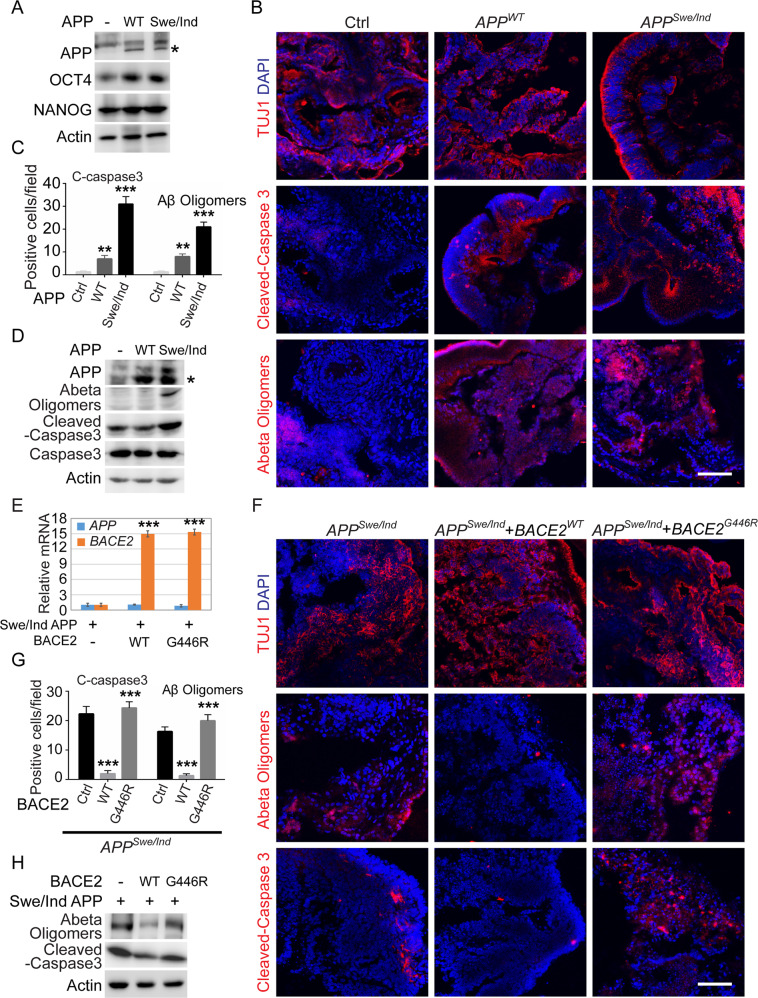
BACE2 overexpression in BOs with family AD mutations attenuates AD-associated phenotypes. **A** Western blot analyses of total proteins from hPSCs stably overexpressed *WT* and *APP* ^*Swe/Idl*^ using the indicated antibodies. **B**–**D** Representative images and quantification of the cellular apoptosis and Aβ accumulation evaluated by immunostaining and western blotting analysis using the indicated antibodies. Scale bar: 100 μm. **E** qRT-PCR analysis of the mRNA level of *APP* and *BACE2* in hPSCs stably overexpressed with *WT* and *APP* ^*Swe/Idl*^. **F**–**H** Representative images and quantification of the cellular apoptosis and Aβ accumulation evaluated by immunostaining and western blot analysis using the indicated antibodies. Scale bar: 100 μm.

To further validate the protective role of BACE2-mediated APP cleavage in BOs, we generated the *APP*^*WT*^ and *APP*^*Swe/Idl*^ stably overexpressed hPSC lines respectively by infecting PSCs with corresponding lentiviruses. APP^*Swe/Idl*^ was an early AD-associated mutation validated in patients and transgenic mice [23–25]. All these ES cells maintained typical flat colony morphology, and the overall protein level of PSC core pluripotent factors, such as OCT4 and NANOG, was not significantly altered upon APP overexpression (Figs. 4A and S2B). Then the hPSC-derived BOs were utilized for modeling the AD patient-associated phenotypes in the context of *APP*^*WT*^ and *APP*^*Swe/Idl*^ overexpression respectively. As anticipated, TUJ1 immunostaining on day 60 revealed that BOs were normally differentiated and organized, while the organoids carrying the AD patient-associated APP mutations significantly increased the Aβ oligomer accumulation and cleaved caspase3-positive cells (Fig. 4B, C). Consistently, western blotting analyses also showed higher cleaved-caspase3 ratio and Aβ oligomer accumulation levels in *APP*^*Swe/Idl*^ mutant BOs (Figs. 4D and S2C). These results indicated that *BACE2*^*G446R*^ BOs indeed exhibited AD-like phenotypes. We then intend to investigate whether enhancing BACE2 expression ameliorated the AD-like phenotypes in *APP*^*Swe/Idl*^ mutant BOs. To this end, *APP*^*Swe/Idl*^ mutant hPSCs were stably transduced with either *BACE2*^*WT*^ or *BACE2*^*G446R*^, and then these cells were utilized for BO growth and immunostaining analysis (Fig. 4E). The results revealed that *APP*^*Swe/Idl*^ mutant organoids with *BACE2*^*WT*^ overexpression, rather than *BACE2*^*G446R*^, significantly decreased the Aβ oligomer accumulation and cell apoptosis ratios compared with the control organoids (Figs. 4F–H and S2D). These findings suggested that enhancing the BACE2 expression may represent a promising strategy to reduce Aβ accumulation and neural cell death.

## Discussion

**Fig. 5:**
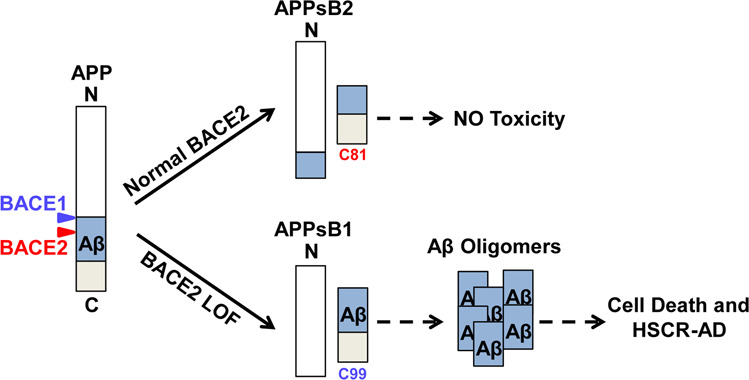
BACE1/BACE2-APP signaling axis in HSCR and AD. Working model for illustrating the deficiency of BACE2-mediated APP cleavage may represent a common pathological mechanism for both HSCR and AD.

In this study, we developed the hPSC-derived BOs and demonstrated the BOs with HSCR-associated BACE2 loss-of-function mutation exhibited AD-like phenotypes, including Aβ accumulation and neuronal cell death, which could also be detected in BOs with familial APP^*Swe/Idl*^ mutations, indicating that dysregulation of BACE2-mediated APP cleavage represents a possible pathogenesis mechanism of AD (Fig. 5). Therefore, specific activation of BACE2 activity in this context may serve as a potential therapeutic strategy for AD treatment.

BACE2 is a homolog of BACE1 and initially considered to be functioned as a β-secretase [26, 27]. However, its contribution to Aβ production could be negligible in comparison with BACE1, owing to the rather weak expression of BACE2 in the brain [28]. In our hPSC-derived BOs, we observed that BACE2 expression level was gradually increased following the maturation of organoids, and its expression was mainly detected in the ventricular zone and cortical plate. These findings are consistent with recently published data that *BACE2* mRNA was expressed in subsets of neurons, oligodendrocytes, and astrocyte-like cells lining the lateral ventricles in the mouse brain detected by in situ hybridization technique [29]. Emerging evidences revealed that BACE2 may function as an alternative α-secretase, and may be not responsible for the pathogenesis of AD, due to the cleavage site of BACE2 is in the Aβ domain [17]. Moreover, some study has identified BACE2 as a potent Aβ-degrading protease based on its high catalytic efficiency to degrade Aβ intracellularly [30]. BACE2 therefore represented a therapeutic candidate for the treatment or prevention of AD. However, all these conclusions are obtained from an in vitro 2D cell culture system. We here combined 3D BO system and HSCR-identified BACE2 loss-of-function genetic variant, and further revealed that BCAE2 played a protective role for Aβ accumulation-induced neuronal cell death. More importantly, Alić et al recently have found that BACE2 was a dose-sensitive AD-suppressor gene in human brains and hPSC-derived BOs, thereby potentially explaining the dementia delay in ~30% of people with Down Syndrome [31], which is consistent with our conclusion that dysregulation of BACE2-mediated APP cleavage may represent a pathological mechanism for AD.

Both of HSCR and AD belong to the genetic diseases resulted from nervous system disorder. However, most of the disease-associated genes are not well documented. Our previous population-based rare variant association study identified BACE2 as a novel HSCR gene. Further clarification of its function using hPSC-based enteric cell model revealed that BACE2 could abolish Aβ production and prevent the accumulation of amyloid to protect neurons from undergoing apoptosis. Here, we further demonstrated that HSCR-associated BACE2 loss-of-function mutation exhibited AD brain-like phenotypes in hPSC-based BOs, indicating that deficiency of BACE2-mediated APP cleavage may represent a common pathological mechanism for both HSCR and AD. Therefore, further validation of the association of HSCR-AD in cohort will be necessary in the future.

## Supplementary information

Uncropped western blots 1

Uncropped western blots 2

Supplementary table 1

Supplementary table 2

## Data Availability

All data generated or analyzed during this study are included in this published article and its Supplementary files and available from the corresponding authors on request.
