# Supplementary figures and images for: BACE2 variant identified from HSCR patient causes AD-like phenotypes in hPSC-derived brain organoids
Source: Cell Death Discov. 2022 Feb 2;8:47. doi: 10.1038/s41420-022-00845-5 (PMC8811022; doi:10.1038/s41420-022-00845-5)

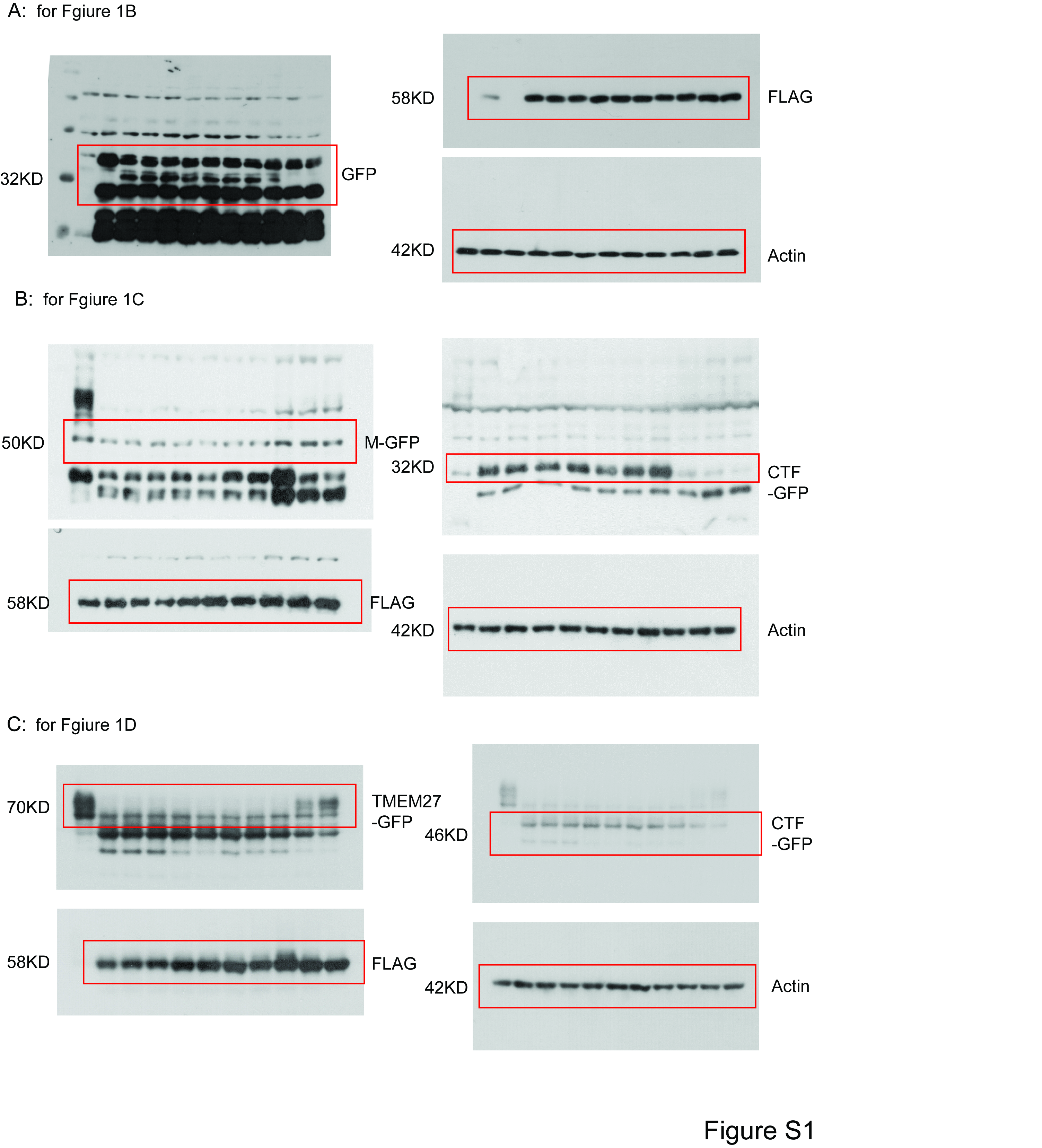

Supplement: Supplementary file 1 — Uncropped western blots 1 [file 41420_2022_845_MOESM1_ESM.tif]

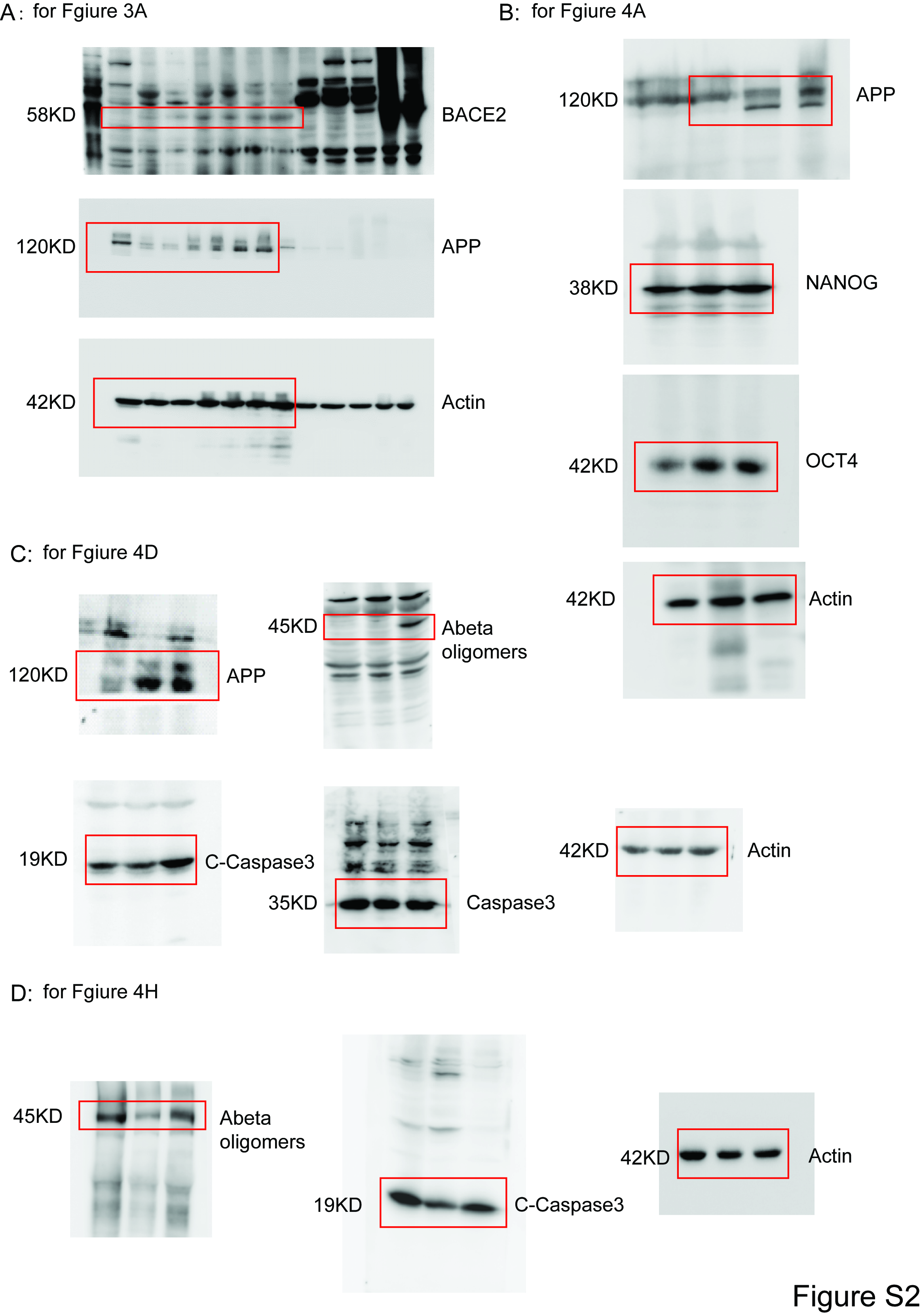

Supplement: Supplementary file 2 — Uncropped western blots 2 [file 41420_2022_845_MOESM2_ESM.tif]
