# Supplementary material for: BACE2 variant identified from HSCR patient causes AD-like phenotypes in hPSC-derived brain organoids
Source: Cell Death Discov. 2022 Feb 2;8:47. doi: 10.1038/s41420-022-00845-5 (PMC8811022; doi:10.1038/s41420-022-00845-5)
Supplement: Supplementary file 3 — Supplementary table 1 [file 41420_2022_845_MOESM3_ESM.docx]

**Supplementary table 1: List of oligos used in this study**

| Target genes | Oligo sequences | Experiment used |
| --- | --- | --- |
| *GAPDH* | Forward: 5'-TCGGAGTCAACGGATTTGGT-3'  Reverse: 5'-TTCCCGTTCTCAGCCTTGAC-3' | qRT-PCR |
| WT *APP* | Forward: 5’-CGGGCATCTGTAAGTGGTTCA-3’  Reverse: 5’-ACCCTTCCATGTGCAGCTTA-3’ | qRT-PCR |
| Mut *APP* | Forward: 5’-ACCTTGAAGCCATTCCTGGG-3’  Reverse: 5’-TCCTGCAGACTTGGCATCAG-3’ | qRT-PCR |
| *BACE2* | Forward: 5’-ATCTCAGAACGGTCAGGCAG-3’  Reverse: 5’-TGAAGGCCCCTTTTTGCTTTG-3’ | qRT-PCR |
