# Supplementary material for: BACE2 variant identified from HSCR patient causes AD-like phenotypes in hPSC-derived brain organoids
Source: Cell Death Discov. 2022 Feb 2;8:47. doi: 10.1038/s41420-022-00845-5 (PMC8811022; doi:10.1038/s41420-022-00845-5)
Supplement: Supplementary file 4 — Supplementary table 2 [file 41420_2022_845_MOESM4_ESM.docx]

**Supplementary table 2: List of antibodies used in this study**

| Target protein | Description | Company  Cat #. | Working concentration | Experiment used |
| --- | --- | --- | --- | --- |
| APP | [KO Validated] APP Rabbit mAb | ABclonal A17911 | 1:1000 | WB |
| BACE2 | Rabbit polyclonal anti-BACE2 | Thermo fisher scientific PA1-754 | 1:1000 | WB |
| Aβ oligomers | Rabbit polyclonal anti-Amyloid oligomers | Abcam ab126892 | 1:1000 | WB |
| Cleaved-caspase3 | Rabbit monoclonal anti-Cleaved caspase-3 (Asp175) (5A1E) | Cell signaling #9664 | 1:1000 | WB |
| Caspase3 | [KO Validated] Casepase-3 Rabbit pAb | ABclonal A2156 | 1:1000 | WB |
| GFP | Goat polyclonal anti-GFP | Rockland  600-101-215 | 1:2000 | WB |
| OCT4 | Oct-3/4(C-10) a mouse monoclonal antibody | Santa cruz sc-5279 | 1:500 | WB |
| NANOG | Anti-NANOG rabbit polyclonal antibody | Sangon Biotech D155241 | 1:1000 | WB |
| Flag | Mouse anti DDDDK-Tag mAb | ABclonal AE005 | 1:3000 | WB |
| β-Actin | ACTB Monoclonal Antibody | ABclonal AC004 | 1:5000 | WB |
| Rabbit IgG | HRP Goat Anti-Rabbit IgG（H+L） | ABclonal AS014 | 1:5000 | WB |
| Mouse IgG | HRP Goat Anti-Mouse IgG（H+L） | ABclonal AS003 | 1:5000 | WB |
| TUJ1 | Mouse monoclonal anti-TUJ1 | Convance PRB-435P | 1:500 | IF |
| Cleaved-caspase-3 | Rabbit monoclonal anti-cleaved caspase-3 (Asp175) (5A1E) | Cell signaling #9664 | 1:100 | IF |
| Amyloid oligomers | Rabbit polyclonal anti-Amyloid oligomers | Abcam ab126892 | 1:100 | IF |
| SOX2 | Goat anti-hSOX2 antibody | R&D AF2018 | 1:250 | IF |
| PAX6 | Rabbit anti-PAX6 antibody | Thermo Fisher  42-6600 | 1:250 | IF |
| FOXG1 | Rabbit anti-FOXG1 antibody | Abcam #18259 | 1:200 | IF |
| PAX2 | Mouse anti-PAX2(194-303) | Abnova H00005076-M01 | 1:200 | IF |
| TBR2 | Rabbit anti-TBR2 antibody | Abcam #23345 | 1:200 | IF |
| TBR1 | Rabbit anti-TBR2 antibody | Abcan #31940 | 1:500 | IF |
| CTIP2 | Rat anti-CTIP2 antibody | Abcam #18465 | 1:100 | IF |
| SATB2 | Rabbit anti-SATB2 antibody | Cell Marque  384R-14 | 1:100 | IF |
| CUX1 | Mouse anti-Cut-like1 antibody | Abcam #242194 | 1:200 | IF |
| REELIN | Mouse anti-Reelin antibody | Millipore MAB5366 | 1:100 | IF |
| GFAP | Rabbit anti-GFAP antibody | Dako Z0334 | 1:200 | IF |
| TH | Mouse anti-tyrosine hydroxylase (TH) | Chemicon MAB318 | 1:200 | IF |
| 5HT | Rat anti-Serotonin antibody | Abcam #6336 | 1:100 | IF |
| vGlut2 | Mouse anti-vGlut2 antibody [8G9.2] | Abcam #79157 | 1:100 | IF |
